# Efficacy of MUC1-targeted CAR-NK cells against human tongue squamous cell carcinoma

**DOI:** 10.3389/fimmu.2024.1337557

**Published:** 2024-02-07

**Authors:** Xiaolan Lin, Tian Guan, Yun Li, Yanchun Lin, Guowei Huang, Yan Lin, Pingnan Sun, Congzhu Li, Jiang Gu, Haoyu Zeng, Changchun Ma

**Affiliations:** ^1^ Department of Radiation Oncology, Cancer Hospital of Shantou University Medical College, Shantou, Guangdong, China; ^2^ Guangdong Procapzoom Bioscience Inc., Guangzhou, China; ^3^ Procapzoom - Shantou University Medical College induced pluripotent stem cell (iPS) Research Center, Shantou, Guangdong, China; ^4^ Guangdong Provincial Key Laboratory of Infectious Diseases and Molecular Immunopathology, Shantou University Medical College, Shantou, Guangdong, China; ^5^ Department of Medical Imaging, the Second Affiliated Hospital, Shantou University Medical College, Shantou, Guangdong, China; ^6^ Department of Stem Cell Research Center, Shantou University Medical College, Shantou, Guangdong, China; ^7^ Department of Gynecological Oncology, Cancer Hospital of Shantou University Medical College, Shantou, Guangdong, China; ^8^ Key Laboratory of Molecular Target & Clinical Pharmacology, School of Pharmaceutical Sciences, Guangzhou Medical University, Guangzhou, Guangdong, China; ^9^ State Key Laboratory of Respiratory Disease, School of Pharmaceutical Sciences, Guangzhou Medical University, Guangzhou, Guangdong, China; ^10^ Guangdong Provincial Key Laboratory for Breast Cancer Diagnosis and Treatment, Cancer Hospital of Shantou University Medical College, Shantou, Guangdong, China

**Keywords:** iPSC-derived MUC1-targeted CAR-NK cells, oral tongue squamous cell carcinoma, mucin 1, chimeric antigen receptor (CAR), induced pluripotent stem (iPS) cell, NK cells therapy

## Abstract

**Introduction:**

The clinical efficacy of CAR-NK cells against CD19-expressing blood cancers has been demonstrated, and they have shown potential for treating solid tumors as well. However, the efficacy of CAR-NK cells for treating human oral tongue squamous cell carcinoma (OTSCC) has not been examined.

**Methods:**

We assessed MUC1 expression in human OTSCC tissue and a cell line using immunohistochemistry and immunofluorescence. We constructed NK cells that express CAR targeted to MUC1 from pluripotent stem cells (iPSC-derived MUC1-targeted CAR-NK cells) and evaluated their effectiveness against OTSCC *in vitro* using the xCELLigence Real-Time Cell Analysis system and CCK8 assay, and *in vivo* by measuring xenograft growth daily in BNDG mice treated with MUC1-targeted CAR-NK cells. As controls, we used iPSC-derived NK cells and NK-free media, which were CAR-free and blank, respectively.

**Results:**

MUC1 expression was detected in 79.5% (66/83) of all OTSCC patients and 72.7% (24/33) of stage III and IV. In stage III and IV MUC1 positive OTSCC, 63.6% (21/33) and 48.5% (16/33) patients had a MUC1-positive cancer cell rate of more than 50% and 80%, respectively. The iPSC-derived MUC1-targeted CAR-NK cells exhibited significant cytotoxicity against MUC1-expressing OTSCC cells *in vitro*, in a time- and dose-dependent manner, and showed a significant inhibitory effect on xenograft growth compared to both the iPSC-derived NK cells and the blank controls. We observed no weight loss, severe hematological toxicity or NK cell-mediated death in the BNDG mice.

**Conclusion:**

The MUC1-targeted CAR-NK cells had significant efficacy against human OTSCC, and their promising therapeutic response warrants further clinical trials.

## Introduction

Immunotherapy utilizing genetically engineered immune-cells, such as CAR-T and CAR-NK cells, has demonstrated exceptional clinical efficacy against hematologic malignancies and have shown potential for treating solid tumors as well. This is due to the fact that solid tumors express membrane antigens that are specifically targeted by the corresponding engineered CARs (1, 2).

The FDA recently approved CD19-targeting CAR-T for the treatment of CD19 expressing B cell cancers, like acute lymphoblastic leukemia and non-Hodgkin lymphomas, as well as BCMA-targeted CAR-T for multiple myeloma (3, 4). Furthermore, early phase clinical trials found that anti-CD22 chimeric receptor T cells exhibited similar antitumor effects as CD19-targeted therapies and had the potential to be an alternative treatment for relapsed or refractory pre-B-ALL (5). CAR-T-cell therapy has been associated with severe clinical adverse events, including cytokine release syndrome (CRS) and immune effector cell-associated neurotoxicity syndrome (ICANS) (6–8). In clinical trials that led to the approval of CD19-targeted CAR-T-cell therapy in phase I and phase II, CRS and neurologic events occurred in 58-92% and 21-67% of patients, respectively (9, 10). Progress in CAR-T research against solid tumors has been sluggish because of various challenges, including identifying highly effective antigen targets specifically expressed in tumor cell membranes, and overcoming immunosuppressive tumor microenvironments. Studies have shown that patients receiving CAR-T cell therapy were prone to relapse, mainly from the loss of the antigen or the CAR-T cell exhaustion (2, 11). Due to these hurdles, engineered NK cells armed with NK-tailored CAR constructs (CAR-NK) could offer an alternative option for anticancer immunotherapy.

NK cells are cytotoxic innate lymphocytes that distinguish malignant cells from healthy ones by recognizing out-of-balance activating and inhibitory receptors on the cell surface. Besides their cytotoxic capacity, NK cells can also secrete various cytokines, chemokines, and growth factors that stimulate other immune cells, such as dendritic cells and macrophages, to promote inflammation in the tumor microenvironment and kill malignant cells without discrimination based on presented neoantigens. Unlike T cells, NK cells have a wide range of sources, broader antigenic spectra, higher affinity, faster immune responses, and can attack tumors without antigen-specific receptors or human leukocyte antigen (HLA) matching. This makes them ideal candidates for allogenic sources, with a lower likelihood of causing graft-versus-host disease, as well as more likely to become off-the-shelf products. Research has shown that higher levels of NK cell infiltration and higher expression of genes encoding NK cell receptors, related cytokines, ligands, as well as effector molecules, indicate better outcomes (12, 13). Recently, CAR-transduced natural killer cells achieved curative efficacy against CD19-expressing lymphoid tumors with no observed cytokine release syndrome, neurotoxicity, or graft-versus-host disease (1). In an alternative study on human epidermal growth factor receptor 2 (HER2)-specific CAR-NK cells against HER2-expressing malignancies, HER2 CAR-NK cells demonstrated stronger cytotoxicity than HER2 CAR-T cells without on-target off-tumor effects, and maintained their cytotoxic activity in the immunosuppressive microenvironment (14).

Human OTSCC is the most commonly diagnosed head and neck squamous cell carcinoma (HNSCC) with unfavorable prognosis due to advanced stages at diagnosis and a lack of established early screening methods. Traditional treatments for advanced stages of OTSCC, including surgical resection, adjuvant radiotherapy, or chemoradiotherapy, combined with T cell immune checkpoint inhibitors (such as nivolumab and pembrolizumab recommended as second-line treatment of patients with recurrent/metastatic HNSCC refractory to platinum-based therapy), have yielded less than 50% 5-year overall survival rates. Therefore, there is a significant need to identify novel diagnostic or therapeutic biomarkers and develop new treatments to improve prognosis (15, 16).

MUC1 is a transmembrane O-glycosylated protein expressed on the apical surface of epithelial cells. The overexpression of MUC1 has been associated with the progression of some malignant diseases and poor prognosis (17–21). However, no report exists of MUC1-targeted CAR-NK for OTSCC treatment.

Our preliminary data showed that MUC1 is specifically expressed in human OTSCC. Following this, we constructed iPSC-derived MUC1-targeted CAR-NK cells and tested their efficacy against human OTSCC *in vitro* and in a BNDG mouse xenograft model, providing evidence for the potential clinical trials of CAR-NK cells in solid tumors.

## Materials & methods

### Human OTSCC tissues samples

Pathological tissue samples of 83 OTSCC patients, received surgery from June 2015 to November 2018, were obtained from the Department of Pathology, the Cancer Hospital of Shantou University Medical College (SUMC). The pTNM staging were determined under the guidance of oral cancer AJCC 8th edition guidelines. The patients’ clinical-pathological characteristics are detailed in **Table 1**.

**Table 1:** Correlation between the clinical parameters and MUC1 expression in oral tongue squamous cell carcer tissues.

Clinical parameters		cases	MUC1 expression			P-value
			Positive (%)	Negative (%)	c^2^	
Gender	Male	51	28(54.9)	23(45.1)	2.391	0.122
	Female	32	23(71.9)	9(28.1)		
Age	>=60	36	17(47.2)	19(52.8)	5.429	**0.020**
	<60	47	34(72.3)	13(27.7)		
Depth of invasion(T)	T1	29	22(75.9)	7(24.1)	3.910	**0.048**
	T2+T3+T4	54	29(53.7)	25(46.3)		
Nodal metastasis(N)	N0	65	43(66.2)	22(33.8)	2.804	0.094
	N1,2,3	18	8(44.4)	10(55.6)		
TNM stage	I+II	50	34(68.0)	16(32.0)	2.280	0.131
	III+IV	33	17(51.5)	16(48.5)		

The results in bold are statistically significant.

### Immunohistochemistry

The study utilized human oral tongue squamous cell carcinoma (OTSCC) tissue that was fixed in 4% paraformaldehyde and embedded in paraffin, and subsequently sectioned into 4μm slices. After dewaxing and rehydration, the slides underwent a 20-minute incubation in 3% hydrogen peroxide to block endogenous peroxidase activity. Antigen retrieval was achieved by heating the tissue sections in boiling 0.01 M citrate buffer (pH=6.0) in a high-pressure cooker for 20 minutes, followed by cooling to room temperature. The tissue sections were incubated with primary antibodies overnight at 4°C following a 1-hour incubation with 10% horse serum. Specifically, mouse anti-MUC1 polyclonal antibody (Abcam, Cat#ab22711), rabbit anti-CD56 polyclonal antibody (Proteintech, Cat#14255-1-AP), and rabbit anti-CD16 polyclonal antibody (Proteintech, Cat#16559-1-AP) were used as primary antibodies. As a negative control, parallel slides were incubated with PBS. After primary antibody application and rinsing, the tissue sections were incubated with a secondary antibody (ZSBIO, Cat#PV9000) that reacts with both rabbit and mouse immunoglobulins conjugated with peroxidase at 37°C for 40 minutes. Between every step, the sections were rinsed with 0.01 M PBS 3 times for 5 minutes each. A positive signal was visualized with AEC (ZSBIO, Cat#ZLI-9036), producing a red staining signal. The slides were counterstained lightly with hematoxylin, dehydrated and mounted. To assess staining levels, three senior pathologists scored the stained slides based on the color development’s intensity on a four-tier scale ranging from 0 (absent) to 3 (strong staining) within most cells in three high-power fields (HPF; 40×) per slide in a blinded manner. The scores were then multiplied by their respective percentage of cells, and a score greater than 180 was considered positive.

### Immunofluorescence

The cells slides were washed twice with PBS and then fixed with 4% paraformaldehyde at 4°C for 30 minutes. Subsequently, the paraformaldehyde was discarded, and the slides were blocked with 10% horse serum for 1 hour. After the blocking process was completed, the slides were incubated with rabbit anti-MUC1 polyclonal antibody (BOSTER, USA, Cat#BA1127) at a 1:100 dilution at 4°C overnight. After incubation, unbound primary antibody was washed away with PBS, and the slides were incubated with goat anti-rabbit antibody with fluorescence 488nm at room temperature for 2 hours inside an opaque box. To examine non-specific binding, PBS was added as a negative control. Subsequently, the slides were captured with a ZEISS Axio Imager A2 fluorescence microscope and mounted.

### MUC1-CAR construction and NK cell differentiation

The MUC1-CAR was fabricated by combining the extracellular domain containing MUC1 scFv sequence, transmembrane region, intracellular domain 4‐1BB, and CD3ζ signal peptide. The corresponding sequence was cloned on the pWXLD lentiviral vector, embedded in lentivirus, and transfected into HEK 293T cells. Transfection of HEK 293T cells was initiated using Opti-MEM medium supplemented with 1 µg/mL pMD.2G, 2 µg/mL psPAX2, and 10 µg/mL PEI. After about 6 hours of transfection, the medium was substituted with high glucose DMEM and the supernatant was collected every 24 hours. Lentivirus was extracted from the supernatant using virus concentration reagent (Accurate Biology Inc., Hunan, China, Cat #AG51001). iPSCs were reprogrammed from blood-derived peripheral blood mononuclear cells (PBMC) utilizing the CoMiP system (22). iPSCs were evenly distributed into 12‐well plates at 1-3 × 10^6^ cells per well. The lentiviral vectors were added to the 12‐well plate with 8 µg/mL of polybrene. After 24 hours, iPSCs were returned to the original medium. The procedure for inducing differentiation of CAR-iPSCs/iPSCs into iPSC-derived CAR-NK/NK cells followed the STEMdiff™ NK Cell Kit (STEMCELL, Cat #100-0170) guideline. In brief, CD^34+^ hematopoietic progenitor cells were first generated from the iPSCs using the STEMdiff™ NK Cell Kit. iPSCs were seeded in AggreWell™ 400 6-well plates (STEMCELL, Cat #34421) and cultured with EB formation medium (STEMCELL). Half of the medium was replaced with EB medium A on day 2, transferred to EB medium B on day 3, and CD^34+^ hematopoietic progenitor cells were harvested on day 12. The isolated CD^34+^ hematopoietic progenitor cells were then seeded in coated AggreWell™ 400 24-well plates (STEMCELL, Cat #34411) and cultured with StemSpan™ Lymphoid Progenitor Expansion Medium for roughly two weeks. On day 14, the medium was altered to StemSpan™ NK Cell Differentiation Medium (STEMCELL) and cultured for an additional two weeks. After that, on day 28, iPSC-derived CAR-NK/NK cells were ready for harvest.

In this experiment, the concentration of the lentivirus used was 1.3 x10^8^ TU/mL. To determine this concentration, we employed the serial dilution method with Jurkat E6 clone cells. Specifically, we performed a gradient dilution of the lentivirus and subsequently measured the positive ratio after 48 hours. We then selected cells within the range of 5% to 20% positive ratio to calculate the lentivirus concentration using the following formula: Concentration = (DL * PR * Q)/V. Here, DL represents the level of dilution, PR represents the positive ratio, Q represents the quantity of Jurkat E6 clone cells seeded, and V represents the volume of the liquid used for transduction. The range of the Multiplicity of Infection (MOI) in our experiments was from 2 to 5. The best suggested MOI we used was 3. In reference to the transduction of iPSCs, we have carefully refined and augmented the existing lentiviral transduction protocol (23). Specifically, 3μL of Polybrene (8 mg/mL) was introduced into 12μL of lentivirus having a concentration of 1.3 x10^8^ TU/mL. Following a 5-minute incubation period, the resultant complex was seamlessly incorporated into 3mL of transduction medium housing 5 × 10^5^ iPS cells within a 6-well plate. Upon successful transduction, the proportion of MUC1-CAR expression in induced pluripotent stem cells (iPSCs) can surpass 90% and under optimal conditions may even approach 99%, particularly when subjected to selection through flow sorting. Nevertheless, post differentiation from CAR-iPSC to CAR-NK cells, a slight reduction of approximately 10% in the positive ratio of MUC1-CAR is observed. Consequently, the positive ratio of MUC1-CAR in MUC1-NK cells consistently maintains a level of approximately 80% during the process of cultivation and subsequent injection into the mouse model.

### Human OTSCC culture

Human OTSCC cell line (TSCCa) cells were cultured in RPMI-1640 supplemented with 10% heat-inactivated fetal bovine serum (FBS; Tianhang Inc., Zhejiang, China), 1% L-glutamine, 1% sodium pyruvate, 50 U/mL penicillin, and 50 μg/mL streptomycin in a 37°C incubator with 5% CO_2_. The cells were routinely evaluated for mycoplasma contamination.

### Cell viability assessment with RTCA instrument

The non-invasive killing effects of MUC1-CAR-NK cells against TSCCa were evaluated in a time-dependent manner using the xCELLigence RTCA instrument (Agilent, Santa Clara, USA) at a temperature of 37°C and 5% CO_2_. RPMI-1640 with 10% FBS was administered to an E-Plate 16 (Agilent) at 50 μL per well to calculate the background impedance. Furthermore, 100 μL of TSCCa cell suspension in RPMI-1640 with 10% FBS was added to each well at a density of 1 × 10^5^ cells/mL. Impedance was recorded every 15 minutes for approximately 22 hours. For the corresponding experimental group, the RPMI-1640 in E-Plate 16 wells was half-replaced with either NK-culturing medium for the blank control group or with iPSC-derived MUC1-targeted CAR-NK cells and iPSC-derived NK cells suspension. Impedance was calculated every 15 minutes for about 24 hours in a continuous manner. The NK cell suspension was diluted to a density of 5 × 10^4^ cells/mL before co-culturing with the TSCCa cells. Subsequently, the cell index of each group measured by the xCELLigence RTCA was normalized to the control group throughout the entire timeline.

### Cell viability detection with the cell counting kit-8 assay

To assess the viability of human OTSCC cell line (TSCCa cells), CCK8 protocol was employed in a dose-dependent manner. TSCCa cells (1 × 10^5^ cells/mL) were initially grown in 96-well plates (100 μL per well) and allowed to adhere for 24 hours. The next day, iPSC-derived MUC1-targeted CAR-NK cells and iPSC-derived NK cells suspension were added to the MUC1-CAR and the MUC1-CAR free NK groups, respectively, in a dose-dependent manner. The target cells to effector cells’ ratios were 1:1, 1:3, and 1:10. The blank control group was supplemented with NK medium only. Following a 12-hour incubation, the NK culturing medium and NK cell suspension were replaced with fresh medium. Afterward, CCK8 reagent (10 μL) was added dropwise to each well, and the plate was incubated for 2-4 hours in the dark. Optical density (OD) was measured at a 450 nm wavelength by an enzyme labeling instrument (Potenov Inc., Beijing, China, Cat #PT-3502B).

### Efficacy of MUC1-targeted CAR-NK cells on OTSCC xenograft mouse model

**Figure 1:**
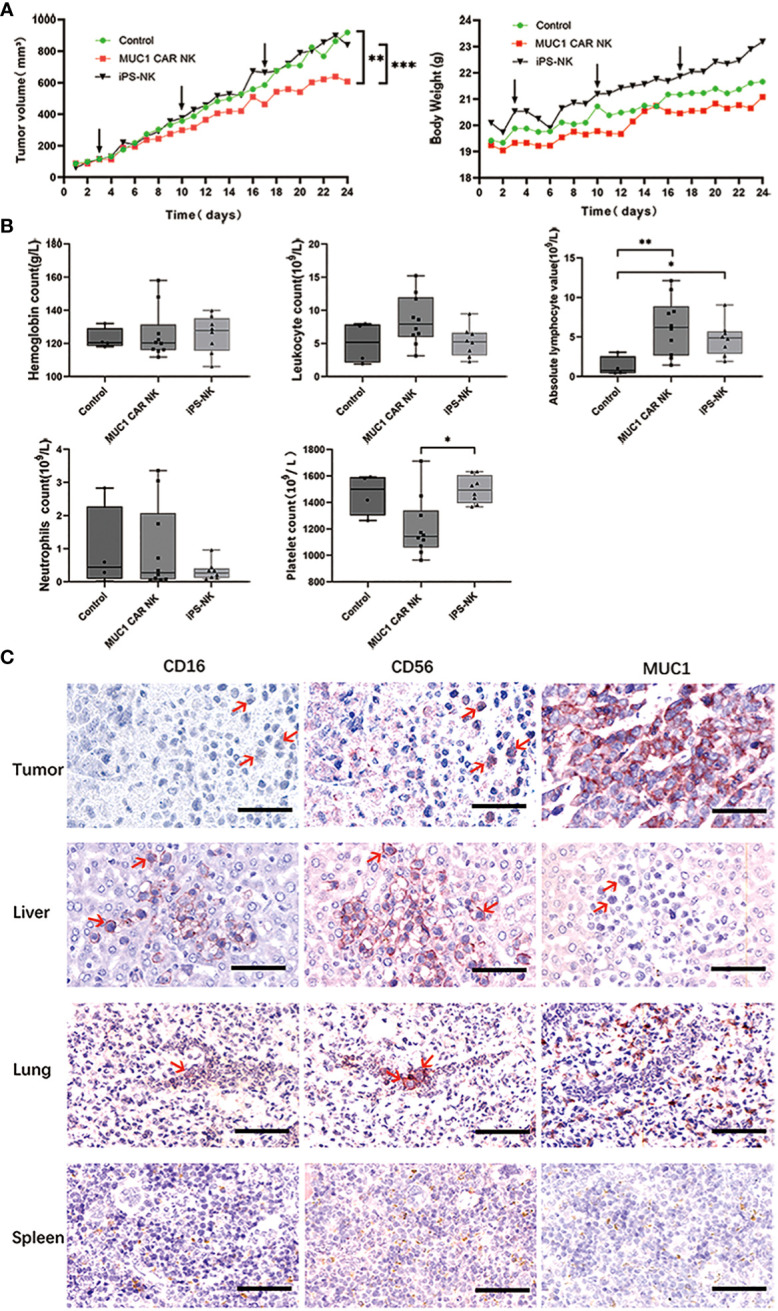
iPSC-derived MUC1-targeted CAR-NK cells restrict tumor growth in OTSCC xenograft mice without causing significant toxic side effects. **(A)** Tumor volume growth curves of OTSCC xenograft mice treated with iPSC-derived MUC1-targeted CAR-NK cells, iPSC-derived NK cells, and normal saline respectively (A left). Mouse weight changes in different treatment groups (A right). Black arrow represents the time of administration. The difference of each group (n = 10/group) was analyzed by two-way ANOVA and Sidak *t* test. **P < 0.01, ***P < 0.001. **(B)** Blood routine results of OTSCC xenograft mice after treatment with normal saline (n=4), iPSC-derived MUC1-targeted CAR-NK (n=10), iPSC-derived NK cells (n=8). *P < 0.05, **P < 0.01. One-way ANOVA and Tukey’s test. **(C)** IHC staining with primary antibodies to CD16, CD56 and MUC1 on sections of the tumor, liver, lung and spleen of a representative OTSCC xenograft mouse from the iPSC-derived MUC1-targeted CAR-NK cells treatment group. Scale bar: 50μm.

We selected the NOD-Prkdc^scid^ lL2rg^tm1^/Bcgen (BNDG) mouse as our *in vivo* xenograft model due to its non-obese diabetes (NOD-scid), severe combined immunodeficiency background, and lack of common receptor subunits for IL2, IL-4, IL-7, IL-9, IL-15, and IL-21 (IL2rg^null^). This choice provides several advantages, including easier establishment of human cancer xenografts and transplantation of human immune cells, human peripheral blood mononuclear cells, ES and iPSC over nude or NSG mice. We obtained the mice from Biocytogen Pharmaceuticals (Beijing) Co., Ltd (Jiangsu, China) and housed them in the specific pathogen-free (SPF) breeding unit of the Experimental Animal Center of Shantou University Medical College. To establish the OTSCC cell line xenograft model, we subcutaneously injected 4 × 10^6^ OTSCC cells in 100 μL of PBS into each mouse’s right flank. On the first day of experimentation, the median tumor volume was observed to be approximately 50 mm³. Subsequently, by the time we initiated administration of NK cells on the third day, the median tumor volume had escalated to 100 mm³. The mice were subsequently randomly divided into 3 groups of 10 mice each. To highlight the commencement of NK cell treatment, we have prominently indicated the administration time with black arrows in **Figure 1A**. We injected 100 μL of PBS suspension containing 1 × 10^7^ iPSC-derived MUC1-targeted CAR-NK cells and iPSC-derived NK cells through the tail vein of each mouse at a weekly interval for three consecutive weeks. The control group was injected with 100 μL of normal saline. We measured and recorded the maximum longitudinal diameter of the tumor as the length, and the maximum transverse diameter perpendicular to the length as the width. We calculated the tumor volume using the formula: tumor size (mm3) = ½ (length × width^2^) each day. We used an electronic balance with an accuracy of 0.001g to weigh and record the mice’s weight daily. After one month, we euthanized all mice and collected approximately 0.5 ml of blood from their retrobulbar vessels for blood routine examination. We fixed the subcutaneous tumor, liver, spleen, and lung with paraformaldehyde, embedded them in paraffin, and sectioned them for immunohistochemical staining.

### Statistical analysis

We performed statistical analysis of all data using GraphPad Prism 8.0 and IBM SPSS Statistics 25 software. We utilized the chi-square test to analyze data for categorical variables. Univariate and multivariate analyses of prognostic factors were accomplished using Cox proportional hazards regression models. For pairwise comparisons between groups of data, we used one-way ANOVA and the Tukey’s test. To analyze the differences in tumor size and body weight of mice in different groups, we performed Two-way ANOVA and Sidak t test. A p-value of less than 0.05 was regarded as statistically significant.

## Results

**Figure 2:**
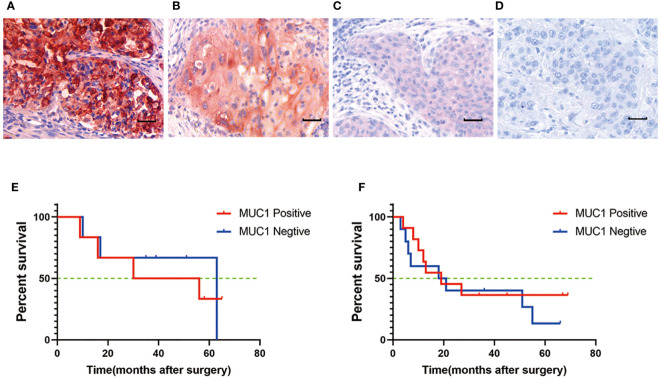
MUC1 expression in human oral tongue squamous cell cancer. **(A)** Strong positive staining of MUC1. Positive staining of MUC1 was mainly located on the membrane and in the cytoplasm of cancer cells. **(B)** Moderate positive staining of MUC1. **(C)** Weak positive staining of MUC1. **(D)** Negative control. Scale bar: 30 µm. There was no significant difference of overall survival between MUC1 positive and MUC1 negative OTSCC patients either in stage III (P=0.07) **(E)** or in stage IV (P=0.32) **(F)**. Kaplan–Meier method and log-rank test.

The study revealed that MUC1 staining was specifically detected in 79.5% of all human OTSCC tissue sections (66/83) and in 72.7% (24/33) of stage III and IV patients. Among MUC1-positive OTSCCs in stage III and IV, 63.6% (21/33) and 48.5% (16/33) of patients had MUC1-positive cancer cell rates of more than 50% and 80%, respectively. Cox regression analysis identified pTNM stages and age, but not MUC1 expression, as independent prognostic predictors in OTSCC patients receiving surgery. The characteristics of the OTSCC patients are detailed in **Tables 1** and **2**, and MUC1 expression did not show a significant correlation with different stages of the patients. MUC1 staining was detected with different intensities on the membrane and in the cytoplasm of OTSCC cells (**Figures 2A–C**), while a negative control was established by replacing the primary antibody with PBS (**Figure 2D**). Overall survival analysis did not reveal any significant difference between MUC1-positive and MUC1-negative OTSCCs, either in stage III (**Figure 2E**) or stage IV (**Figure 2F**). In our investigations, we did not observe specific MUC1 staining in cell types within human OTSCCs, as well as in various human tissues including liver, cerebrum, stomach, heart, and skeletal muscle, as determined from autopsy specimens. However, slight MUC1 staining was detected in the spleen, kidney, and colon (**Supplementary Figure 1**). It is worth noting that the Human Protein Atlas (available at https://www.proteinatlas.org/ENSG00000185499-MUC1/pathology) indicates the presence of MUC1 in several human tissues, particularly in the kidney and stomach. This inconsistency in MUC1 expression may arise from interindividual variation in MUC1 expression levels and the utilization of diverse MUC1 detection techniques.

**Table 2:** Univariate and multivariate analysis of prognostic factors in patients with OTSCC.

Clinical Parameters	Uni-variate			Multi-variate		
	P-value	Hazard ratio	95% CI	P-value	Hazard ratio	95% CI
Age (years)						
< 60/≥ 60	**0.007**	0.362	0.173-0.759	**0.004**	0.333	0.159-0.699
Depth of invasion						
T_1 + 2_/T_3 + 4_	**0.001**	0.287	0.140-0.589	0.540	1.501	0.410-5.497
Nodal metastasis						
N_0_/N_1 + 2+3_	**0.001**	0.300	0.145-0.620	0.845	0.907	0.340-2.417
TNM stage						
S_I+II_/S_III+IV_	**0.000**	0.202	0.093-0.440	**0.000**	0.190	0.087-0.415
MUC1 IHC						
Negative/Positive	**0.046**	2.057	1.014-4.173	0.899	1.049	0.498-2.209
Gender						
Male/Female	0.161	1.727	0.845-3.529	0.163	1.356	0.884-2.080

The results in bold are statistically significant.

**Figure 3:**
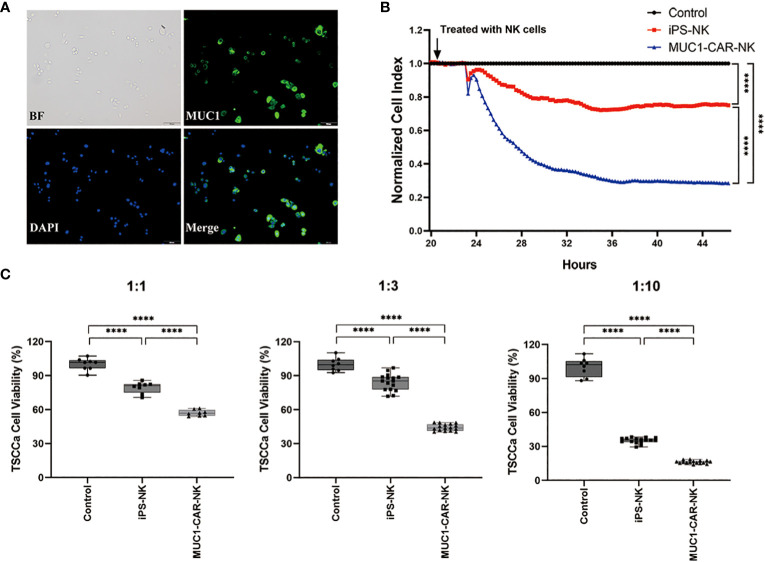
Efficacy of the iPSC-derived MUC1-targeted CAR-NK cells against the MUC1-expressing human OTSCC cell lines. **(A)** Immunofluorescence of TSCCa cells. MUC1 positively expressed on TSCCa cell membrane. **(B)** Time curve of TSCCa cells killed by iPSC-derived MUC1-targeted CAR-NK cells detected by xCELLigence RTCA. Cell viability of TSCCa was calculated by xCELLigence and presented as Cell Index. ****P < 0.0001, One-way ANOVA and Tukey test. **(C)** The efficiency of iPSC-derived MUC1-targeted CAR-NK cells killing TSCCa cells in different ratio of cell concentration *in vitro* condition. Experiments were performed at the ratio of target cells to effector cells corresponding to 1:1, 1:3 and 1:10 respectively. ****P < 0.0001, One-way ANOVA and Tukey’s test.

We employed a preclinical *in vitro* model to evaluate the efficacy of iPSC-derived MUC1-targeted CAR-NK cells against MUC1-expressing OTSCC cell line (TSCCa). Immunofluorescence was performed to confirm the expression of MUC1 in the TSCCa cell line, with specific positive staining observed on the cytomembrane (**Figure 3A**). The viability of MUC1-expressing TSCCa cells was measured in a time- and dose-dependent manner using xCELLigence RTCA and CCK8 assays following co-culture with iPSC-derived MUC1-targeted CAR-NK cells, iPSC-NK cells, and blank control medium. Through the utilization of xCELLigence RTCA, we observed a gradual and rapid decline in viability of MUC1-expressing TSCCa cells co-cultured with iPSC-derived MUC1-targeted CAR-NK cells compared to TSCCa cells co-cultured with iPSC-NK cells (all P < 0.0001) or NK-culturing medium (all P < 0.0001) (**Figure 3B**). A subsequent CCK8 assay revealed a dose-dependent decrease in viability of TSCCa cells co-cultured with iPSC-derived MUC1-targeted CAR-NK cells (at target-to-effector cell ratios of 1:1, 1:3, and 1:10) compared to TSCCa cells co-cultured with iPSC-NK cells (all P < 0.0001) or NK-culturing medium (all P < 0.0001) (**Figure 3C**).

We established a MUC1-expressing human OTSCC cell line (TSCCa) xenograft BNDG mouse model, in which we examined the effectiveness of iPSC-derived MUC1-targeted CAR-NK cells against MUC1-expressing human OTSCC. The xenograft mice were categorized into three groups (n=30), concerning their body weight and tumor size. We treated the xenografts with MUC1-targeted CAR-NK cells, iPSC-NK cells, or normal saline blank control once weekly for three consecutive weeks. In accordance with the Guidelines for Endpoints in Animal Study Proposals, version 2022 (available at https://oacu.oir.nih.gov/system/files/media/file/2022-04/b13_endpoints_guidelines.pdf), it is stated that the dimension of a tumor in a mouse should not exceed 20 mm. Additionally, the tumor burden should not exceed 10% of the respective body weight. Adhering to these two key principles, we were compelled to terminate our animal experiment within a month, as the tumor burden approached nearly 10% of the body weight in all groups as illustrated in **Figure 1A** (right). The results showed that in comparison to the iPSC-NK cells (P < 0.001) and blank control groups (P < 0.01), the xenograft tumor in the iPSC-derived MUC1-targeted CAR-NK cell group exhibited significantly slower growth (**Figure 1A** left). Moreover, no weight reduction was observed in any of the BNDG mice (**Figure 1A** right).

We diligently adhered to the requirement of tumor burden as specified in the Guidelines for Endpoints in Animal Study Proposals, which prompted us to terminate the animal experiment within one month. In order to comprehensively evaluate the safety profile of CAR-NK cells, we conducted two additional independent experiments to assess their on-target specificity and potential toxicity. As depicted in **Supplementary Figure 2B**, through co-culturing with both OTSCC (MUC1 positive) primary cells and paracancerous normal epithelial cells (MUC1 negative), the MUC1-targeted CAR-NK cells exhibited a remarkable and significantly higher cytotoxicity towards the OTSCC primary cells as compared to the normal control. These findings reinforce the specific targeting ability of CAR-NK cells against MUC1-positive cells, thereby underlining their potential therapeutic efficacy. In **Supplementary Figure 5** (data unpublished), we present survival curves obtained from three groups of BNDG mouse OTSCC (MUC1 positive) xenograft models. These groups were respectively injected with CAR-iPS-NK cells, iPS-NK cells, and the blank control (saline). Through weekly monitoring for a duration of three months, we observed that MUC1-CAR-NK cells significantly prolonged the survival of mice. Intriguingly, neither the iPS-NK cells nor CAR-iPS-NK cells induced any mortality in the mice, as evidenced by their noticeably longer survival compared to the blank control (saline) group.

As depicted in **Figure 1B**, dissimilarities in the blood routine results of the control and treatment groups were observed. When compared to the control groups, iPSC-derived MUC1-targeted CAR-NK treatment group exhibited no noteworthy decline in hemoglobin, number of leucocytes, neutrophil or absolute lymphocytes. Although the platelet counts in the iPSC-derived MUC1-targeted CAR-NK treatment group was 1210.2 ± 212.4×10^9^/L (not less than 977 ± 110.4×10^9^/L), the normal range of platelet count of BNDG, it was considerably lower than the iPSC-derived NK treatment groups, and the reason for this is uncertain. The higher number of absolute lymphocytes in both iPSC-derived MUC1-targeted CAR-NK and iPSC-derived NK groups, compared to the blank group, may be attributed to the exogenous NK cells that were delivered into the blood circulation of the BNDG mice. No severe hematologic toxicities were observed in any of the BNDG mice, and no death due to NK cell therapy occurred.

As illustrated in **Figure 1C**, we carried out immunohistochemical staining of MUC1, CD56, and CD16 on tissue sections of the tumor, liver, lung, and spleen collected from a representative xenograft mouse in the iPSC-derived MUC1-targeted CAR-NK cells group. We observed that CD56 and/or CD16-positive NK cells (**Figure 1C** red arrows) could be effortlessly identified in the interface area between cancer cell clusters and the cancer cell lysis site in the MUC1-expressing xenograft tumor tissue. This phenomenon was also observed in the liver and lung (**Figure 1C**). These observations suggest that the exogenous CAR-NK cells successfully passed through the blood circulation from the mouse tail vein to various organs, including the liver and lung, remained active, migrated into the tumor tissue, and played an essential role in cell lysis against the xenograft tumor tissue.

## Discussion

Due to MUC1 expression specifically in human OTSCC, the feasibility of MUC1-CAR in CAR-T cell trials, the ready availability, safety profile, and emerging-evidenced efficacy in fighting cancer of CAR-NK cells, we initially developed and tested the iPSC-derived MUC1-targeted CAR-NK cells’ cytotoxicity against MUC1-expressing human OTSCC cells *in vitro* and in BNDG mouse xenograft models (24–30). The verified substantial effectiveness of the iPSC-derived MUC1-targeted CAR-NK cells against MUC1-expressing human OTSCC in the preclinical models provides evidence for potential clinical trials of CAR-NK cell therapy for solid tumors.

In this study, MUC1 staining was specifically detectable in 79.5% (66/83) of all human OTSCC tissue sections and 72.7% (24/33) of patients in stages III and IV. Of the MUC1-positive OTSCC patients in stages III and IV, 63.6% (21/33) and 48.5% (16/33) respectively had MUC1-positive cancer cell rates of over 50% and 80%, respectively. No positive MUC1 staining was observed in other cell types of human OTSCC tissue. Cox regression analysis showed that pTNM stages and age, rather than MUC1 expression, were independent prognostic predictors. Actually, MUC1 and many other CAR-combining antigens used in immune cell therapy are tumor-associated antigens (TAA) rather than tumor-specific antigens (TSA). Logically, TAA expression in normal tissue or organs could potentially induce on-target/off-tumor toxicity during CAR-T and CAR-NK treatment. As a result, enhancing the on-target effects while reducing off-tumor toxicity for use remains a crucial problem to solve in immune cell therapy, including CAR-T and CAR-NK. In our study, we validated the specific cytotoxicity (On-Target Effects) of iPSC-derived MUC1-targeted CAR-NK cells against MUC1-expressing human OTSCC cells *in vitro* and *in vivo* using MUC1-CAR-free NK cells and NK cells-free blank medium as controls.

In order to address the specificity of MUC1 targeted CAR-NK cells, we developed a customized recombinant protein, MUC1-scFv-Histag, utilizing MUC1-CAR recognizing fragments (31), Subsequent incubation of this recombinant protein with MUC1-positive OTSCC cells and subsequent detection using Flow Cytometry revealed a significantly higher affinity of these cells towards the MUC1-scFv-Histag compared to MUC1-knockout OTSCC cells, MUC1-scFv negative OTSCC cells, and MUC1-negative normal controls. This validates the successful binding of the recombinant protein to MUC1-positive OTSCC cells (**Supplementary Figure 2A**). Additionally, co-culture experiments involving MUC1 targeted CAR-NK cells and OTSCC primary cells (OPC, MUC1 positive) as well as paracancerous normal epithelial cells (PNEC, MUC1 negative) separately, demonstrated that MUC1 targeted CAR-NK cells had significantly specific cytotoxicity towards OTSCC primary cells, while sparing the normal control cells (**Supplementary Figure 2B**).

In our study, we conducted immunohistochemical staining of MUC1, CD56, and CD16 on tissue sections obtained from a representative xenograft mouse in the iPSC-derived MUC1-targeted CAR-NK cells group. Our observations revealed the presence of CD56 and/or CD16-positive NK cells (indicated by red arrows in **Figure 1C**) in the junctional zone between clusters of cancer cells and the area of cancer cell lysis in the MUC1-expressing xenograft tumor tissue, as well as in the liver and lung. These findings suggest that the adoptively transferred CAR-NK cells successfully localized to solid tumors and exerted their cytotoxic activities. However, it is important to note that the distribution of adoptively transferred CAR-NK cells *in vivo*, as well as potential off-target effects, were not comprehensively evaluated in this experiment. This is due to various factors, including differences in host organisms, time points of injection, quantity of NK cells delivered, depletion of NK cells, and MUC1 expression in other tissues.

To address the on-target distribution of MUC1-targeted CAR-NK cells, we visualized the green fluorescence signal in the heart, lung, liver, kidney, spleen, and xenografts of two mice one day after injection of MUC1-NK cells containing reporting fragments (Luciferase) integrated into the MUC1-CAR construct. The significantly higher levels of green fluorescence observed in the tumors provided evidence of the specific on-target distribution of the MUC1-targeted CAR-NK cells (**Supplementary Figure 3A**). Additionally, we performed immunohistochemical staining using an anti-human CD56 antibody on tumor tissue sections, confirming the accumulation of infiltrated MUC1-NK cells within the MUC1-positive tumors (**Supplementary Figure 3B**).

We fully acknowledge the importance of exercising caution and considering the potential for off-target cytotoxicity, despite the generally lower risk of off-target effects associated with NK-CAR cells due to their non-MHC-restricted recognition. We have conducted experiments to validate the on-target effect of iPSC-derived MUC1-targeted CAR-NK cells. Specifically, we have demonstrated the specific binding of the recombinant protein MUC1-scFv-Histag to MUC1-positive cells (**Supplementary Figure 2A**), as well as the specificity of the killing effect exerted by MUC1-targeted CAR-NK cells (**Supplementary Figure 2B** and **Figure 3**). Considering the potential interaction between MUC1 and NK cells in regulating NK cell function and the expression of NKG2D ligands that effectively activate NK cells (32), it is pertinent to note that our CAR-MUC1 construct is derived from the scFv region, representing only a small portion of the extracellular domain of MUC1. The observed lack of proliferation of MUC1-targeted CAR-NK cells suggests that the CAR-MUC1 construct may not interact reciprocally with MUC1-C.

In order to assess the affinity and avidity functions of the MUC1 antibody in the context of CAR-NK cell cytotoxicity against the OTSCC cell line, we conducted additional experiments. Specifically, we monitored the growth of TSCCa (OTSCC cell line) when treated with MUC1 antibody, iPS-NK cells, and MUC1-NK cells, respectively (**Supplementary Figure 4**). Our observations revealed that blocking MUC1 on TSCCa cells led to a slight inhibition of cell growth compared to the blank control. However, when compared to the MUC1 antibody, both iPS-NK cells and MUC1-NK cells exhibited much more pronounced efficacy in directly killing TSCCa cells.

MUC1-targeted CAR-NK cells were developed from iPSCs, and this method has been a revolutionary tool in the regenerative medicine field. The preparation of iPSCs in this study was a reprograming process conducted from the blood-derived peripheral blood mononuclear cells (PBMCs) referred to as the CoMiP system (22). Using this system, iPSCs can be genetically manipulated and engineered with CARs at the pluripotent stage, thereby establishing permanent, stable, and clonal iPSC-CAR lines that serve as inexhaustible sources of unlimited homogenous CAR-NK cells for the manufacture of multiple master iPSC-CAR cell banks. These banks target a variety of antigens for autoimmune diseases, viral infections, and cancers (25, 33–36). In our study, the engineered, modified iPSC-derived MUC1-targeted CAR-NK cells exhibited a significant cytotoxic effect against the MUC1-expressing human OTSCC cells *in vitro* and significant inhibition of OTSCC xenograft growth in a BNDG mouse model. All the BNDG mice that were used in the experiment did not experience any weight loss, significant hematological toxicity, or NK cell-mediated death. However, these common parameters for toxicity evaluation are not sufficient to reflect the adverse effects of CAR-NK cell therapy. Therefore, carefully-designed clinical trials are necessary to evaluate the toxicity of the iPSC-derived MUC1-targeted CAR-NK cells, using other parameters such as heart, liver, and kidney function, electrolyte levels, and cytokine levels (such as IL6, IL10, etc.).

However, the growth inhibition effect of iPSC-derived MUC1-targeted CAR-NK cells on the xenograft tumors was not as strong as observed *in vitro*. This could be due to several factors, including (1) the relatively high ratio of tumor cells to CAR-NK cells at baseline (2), loss of CAR-NK cells in the mouse’s bloodstream (3), the limited number of CAR-NK cells infiltrating the tumor mass, and (4) the relatively brief period between the initial delivery and sacrifice, considering the requirement of animal ethics for tumors larger than the ones used in this study. Concerns have been raised that the number and activity of NK cells in solid tumor masses could be insufficient to sufficiently treat cancer cells (37). A report revealed that a patient with recurrent multifocal glioblastoma who received multiple intracavitary injections of IL13Rα2-targeted CAR-T cells experienced no significant toxic side effects and obtained a significant response (38). These observations suggest that a localized delivery of CAR-NK cells (such as tumor-local administration or interventional therapy via tumor feeding artery) to improve the number of NK cells in tumor tissue could be a potential strategy to consider. Furthermore, antibody-dependent cellular cytotoxicity (ADCC) is a crucial mechanism of NK cells that is facilitated by the Fc receptor CD16a on human NK cells and is mediated by therapeutic monoclonal antibodies (mAbs). A point mutation of CD16a can prevent this activation-induced surface cleavage. This non-cleavable CD16a variant was engineered into human iPSCs as hnCD16-iNK cells and was functionally mature, exhibiting enhanced ADCC against multiple tumor targets (36). Therefore, combining hnCD16-iNK cells with mAbs to generate a standardized off-the-shelf engineered NK cell therapy with improved ADCC properties could be an alternative strategy to improving the efficacy against lethal solid tumors that are resistant to NK cell-mediated killing.

Although the regulatory landscape for iPSC-derived cell therapies is evolving, ensuring safety and efficacy, as well as addressing concerns related to tumorigenicity, is crucial for regulatory approval. Also, creating NK cells from iPSCs is a complex and resource-intensive process. The differentiation and maturation of iPSCs into functional NK cells often involve multiple steps, including genetic modification and quality control as well as multiple injections, which can contribute to high manufacturing costs.

In conclusion, the preclinical models showed that iPSC-derived MUC1-targeted CAR-NK cells effectively killed MUC1-expressing human OTSCC, indicating their promising therapeutic potential. Therefore, further clinical investigation is warranted to evaluate their efficacy in treating OTSCC patients.

## Data availability statement

The original contributions presented in the study are included in the article/**Supplementary Material**, further inquiries can be directed to the corresponding author/s.

## Ethics statement

The studies involving humans were approved by the Ethics Committee of the Cancer Hospital Affiliated to Shantou University Medical College, and informed consent was obtained from all participants. *In vivo* experiments were conducted with the permission of the Medical Animal Care & Welfare Committee of Shantou University Medical College. The studies were conducted in accordance with the local legislation and institutional requirements.

## Author contributions

XL: Formal analysis, Investigation, Methodology, Writing – original draft, Data curation. TG: Data curation, Formal analysis, Investigation, Methodology, Validation, Writing – review & editing. YuL: Formal analysis, Investigation, Methodology, Validation, Writing – review & editing. YCL: Formal analysis, Investigation, Methodology, Validation, Writing – review & editing. GH: Investigation, Methodology, Validation, Writing – review & editing. YaL: Investigation, Writing – review & editing, Funding acquisition. PS: Investigation, Methodology, Resources, Software, Validation, Writing – review & editing. CL: Investigation, Resources, Supervision, Writing – review & editing. JG: Investigation, Methodology, Resources, Supervision, Writing – review & editing. HZ: Conceptualization, Formal analysis, Funding acquisition, Methodology, Project administration, Resources, Supervision, Writing – original draft, Writing – review & editing. CM: Conceptualization, Formal analysis, Funding acquisition, Investigation, Methodology, Project administration, Resources, Supervision, Writing – original draft, Writing – review & editing.

## Supplementary material

Supplementary Figure 1 IHC staining of MUC1 in the tissue sections of human tongue cancer (Oral tongue squamous cell carcinoma, OTSCC) and various human tissues from autopsy specimens. By performing immunohistochemistry staining of MUC1 on tongue cancer, liver, spleen, cerebrum, kidney, stomach, colon, heart and skeletal muscle, we did not observe specific MUC1 staining in other cell types within human OTSCCs, as well as in various human tissues including liver, cerebrum, stomach, heart and skeletal muscle, as determined from autopsy specimens. However, slight MUC1 staining was detected in the spleen, kidney, and colon (**Supplementary Figure 1**). It is worth noting that the Human Protein Atlas (available at https://www.proteinatlas.org/ENSG00000185499-MUC1/pathology) indicates the presence of MUC1 in several human tissues, particularly in the kidney and stomach. This inconsistency in MUC1 expression may arise from interindividual variation in MUC1 expression levels and the utilization of diverse MUC1 detection techniques. Scale bar: 60μm.

Supplementary Figure 2
**(A)** The result showed the specific combination of the recombinant protein MUC1-scFv-Histag with MUC1-positive cells. By incubating with MUC1-scFv-Histag using MUC1-CAR recognizing fragments and subsequent detection using flow cytometry, MUC1-positive OTSCC cells exhibited significant higher affinity to this recombinant protein, compared with MUC1-knockout OTSCC cells, MUC1-scFv negative OTSCC cells and MUC1-negative normal control respectively. **(B)** The result indicated the specificity of the killing effect of MUC1 targeted CAR-NK cells. By co-cultured with OTSCC primary cells (OPC, MUC1 positive) and paracancerous normal epithelial cells (PNEC, MUC1 negative), the MUC1 targeted CAR-NK cells had significantly cytotoxicity towards OTSCC primary cells, while sparing the normal control cells.

Supplementary Figure 3
**(A)** The result showed the on-target distribution of MUC1-targeted CAR-NK cells. We visualized the green fluorescence signal in the heart, lung, liver, kidney, spleen, and xenografts of two mice one day after injection of MUC1-NK cells containing reporting fragments (Luciferase) integrated into the MUC1-CAR construct. The significantly higher levels of green fluorescence observed in the tumors provided evidence of the specific on-target distribution of the MUC1-targeted CAR-NK cells. **(B)** The result also showed the on-target distribution of MUC1-targeted CAR-NK cells. We performed immunohistochemical staining using an anti-human CD56 antibody on tumor tissue sections, confirming the accumulation of infiltrated MUC1-NK cells within the MUC1-positive tumors.

Supplementary Figure 4 Assessment of the affinity and avidity functions of the MUC1 antibody in the context of CAR-NK cell cytotoxicity against the OTSCC cell line. We monitored the growth of TSCCa (OTSCC cell line) when treated with MUC1 antibody, iPS-NK cells, and MUC1-NK cells, respectively. Our observations revealed that blocking MUC1 on TSCCa cells led to a slight inhibition of cell growth compared to the blank control. However, when compared to the MUC1 antibody, both iPS-NK cells and MUC1-NK cells exhibited much more pronounced efficacy in directly killing TSCCa cells.

Supplementary Figure 5 The iPSC-derived MUC1-targeted CAR-NK cells significantly prolonged the survival of OTSCC xenograft BNDG mice. The survival curves were obtained from three groups of BNDG mouse OTSCC (MUC1 positive) xenograft models. These groups were respectively injected with CAR-iPS-NK cells, iPS-NK cells, and the blank control (saline). Through weekly monitoring for a duration of three months, we observed that MUC1-CAR-NK cells significantly prolonged the survival of mice. Intriguingly, neither the iPS-NK cells nor CAR-iPS-NK cells induced any mortality in the mice, as evidenced by their noticeably longer survival compared to the blank control (saline) group.
