# Supplementary figures and images for: Efficacy of MUC1-targeted CAR-NK cells against human tongue squamous cell carcinoma
Source: Front Immunol. 2024 Feb 7;15:1337557. doi: 10.3389/fimmu.2024.1337557 (PMC10882221; doi:10.3389/fimmu.2024.1337557)

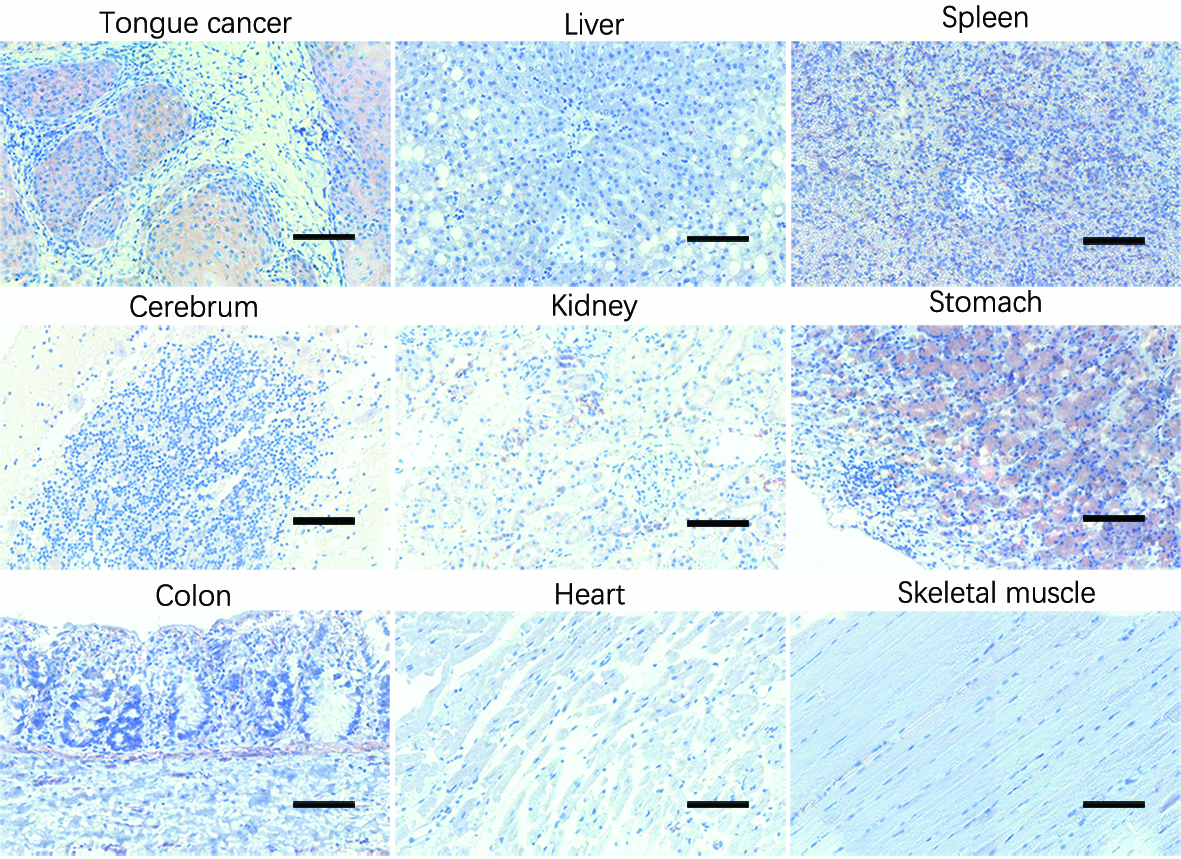

Supplement: Supplementary Figure 1 — IHC staining of MUC1 in the tissue sections of human tongue cancer (Oral tongue squamous cell carcinoma, OTSCC) and various human tissues from autopsy specimens. By performing immunohistochemistry staining of MUC1 on tongue cancer, liver, spleen, cerebrum, kidney, stomach, colon, heart and skeletal muscle, we did not observe specific MUC1 staining in other cell types within human OTSCCs, as well as in various human tissues including liver, cerebrum, stomach, heart and skeletal muscle, as determined from autopsy specimens. However, slight MUC1 staining was detected in the spleen, kidney, and colon ( Supplementary Figure 1 ). It is worth noting that the Human Protein Atlas (available at https://www.proteinatlas.org/ENSG00000185499-MUC1/pathology) indicates the presence of MUC1 in several human tissues, particularly in the kidney and stomach. This inconsistency in MUC1 expression may arise from interindividual variation in MUC1 expression levels and the utilization of diverse MUC1 detection techniques. Scale bar: 60μm. [file Image_1.jpg]

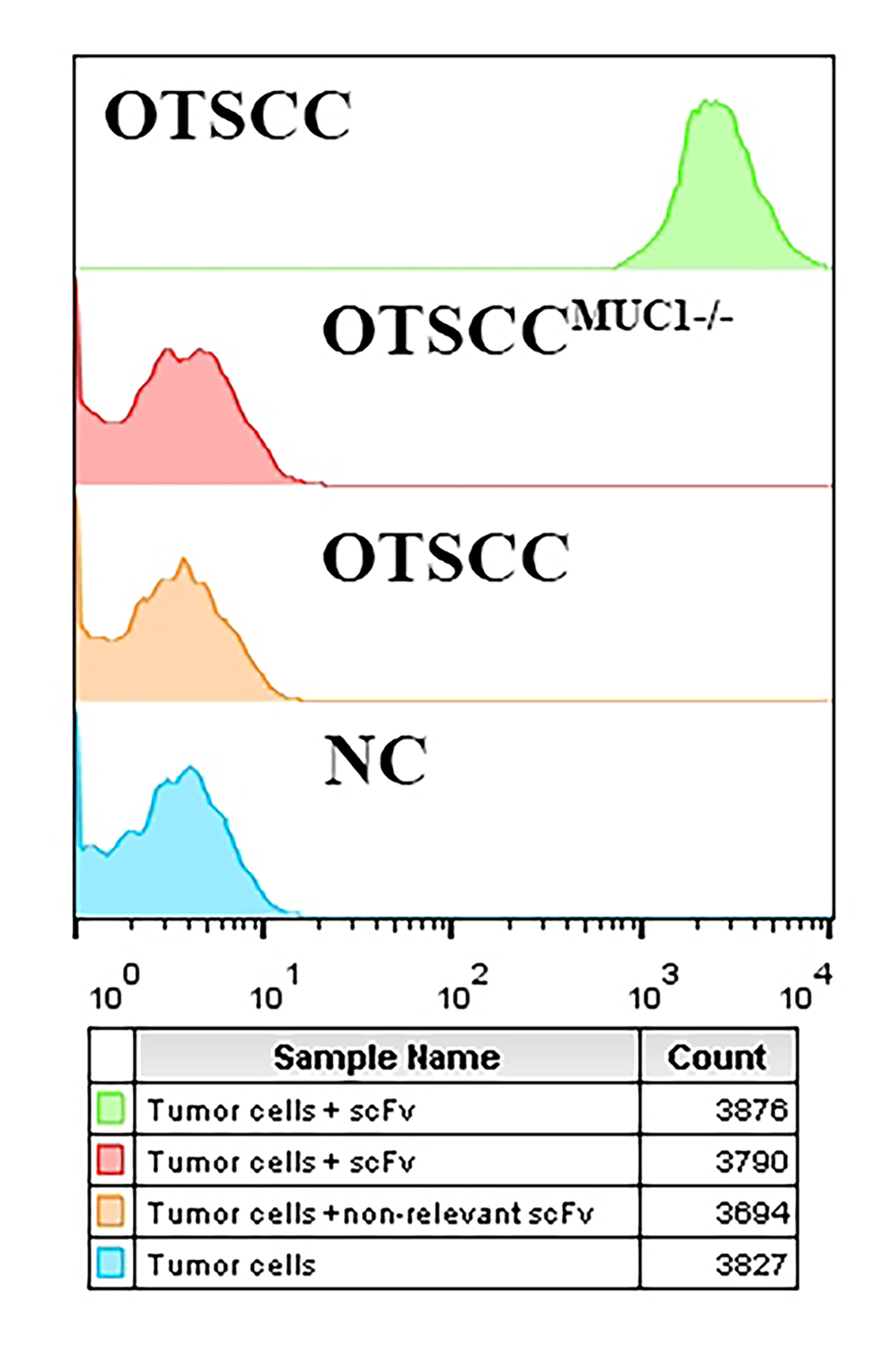

Supplement: Supplementary Figure 2 — (A) The result showed the specific combination of the recombinant protein MUC1-scFv-Histag with MUC1-positive cells. By incubating with MUC1-scFv-Histag using MUC1-CAR recognizing fragments and subsequent detection using flow cytometry, MUC1-positive OTSCC cells exhibited significant higher affinity to this recombinant protein, compared with MUC1-knockout OTSCC cells, MUC1-scFv negative OTSCC cells and MUC1-negative normal control respectively. (B) The result indicated the specificity of the killing effect of MUC1 targeted CAR-NK cells. By co-cultured with OTSCC primary cells (OPC, MUC1 positive) and paracancerous normal epithelial cells (PNEC, MUC1 negative), the MUC1 targeted CAR-NK cells had significantly cytotoxicity towards OTSCC primary cells, while sparing the normal control cells. [file Image_2.jpg]

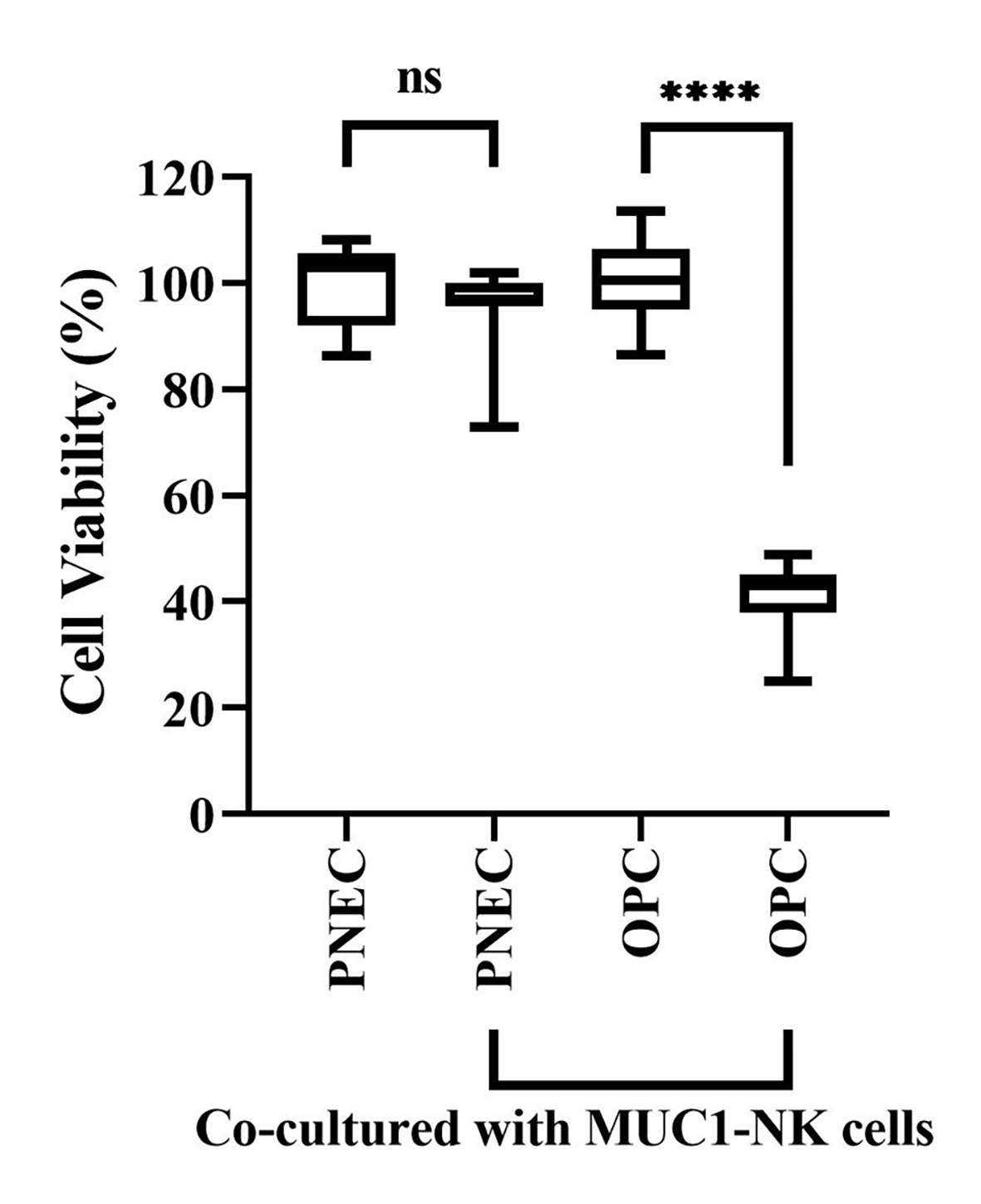

Supplement: Supplementary Figure 3 — (A) The result showed the on-target distribution of MUC1-targeted CAR-NK cells. We visualized the green fluorescence signal in the heart, lung, liver, kidney, spleen, and xenografts of two mice one day after injection of MUC1-NK cells containing reporting fragments (Luciferase) integrated into the MUC1-CAR construct. The significantly higher levels of green fluorescence observed in the tumors provided evidence of the specific on-target distribution of the MUC1-targeted CAR-NK cells. (B) The result also showed the on-target distribution of MUC1-targeted CAR-NK cells. We performed immunohistochemical staining using an anti-human CD56 antibody on tumor tissue sections, confirming the accumulation of infiltrated MUC1-NK cells within the MUC1-positive tumors. [file Image_3.jpg]

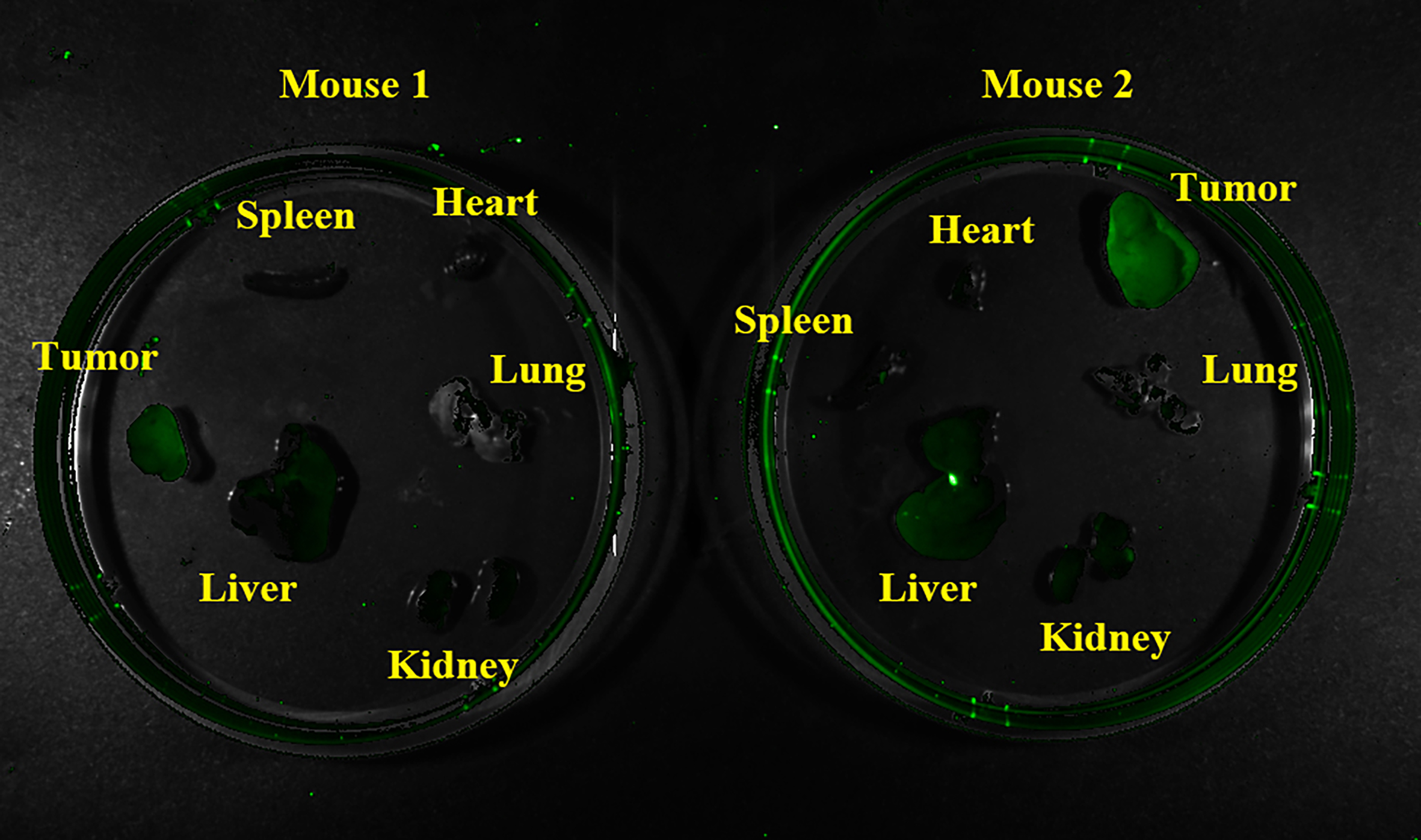

Supplement: Supplementary Figure 4 — Assessment of the affinity and avidity functions of the MUC1 antibody in the context of CAR-NK cell cytotoxicity against the OTSCC cell line. We monitored the growth of TSCCa (OTSCC cell line) when treated with MUC1 antibody, iPS-NK cells, and MUC1-NK cells, respectively. Our observations revealed that blocking MUC1 on TSCCa cells led to a slight inhibition of cell growth compared to the blank control. However, when compared to the MUC1 antibody, both iPS-NK cells and MUC1-NK cells exhibited much more pronounced efficacy in directly killing TSCCa cells. [file Image_4.jpg]

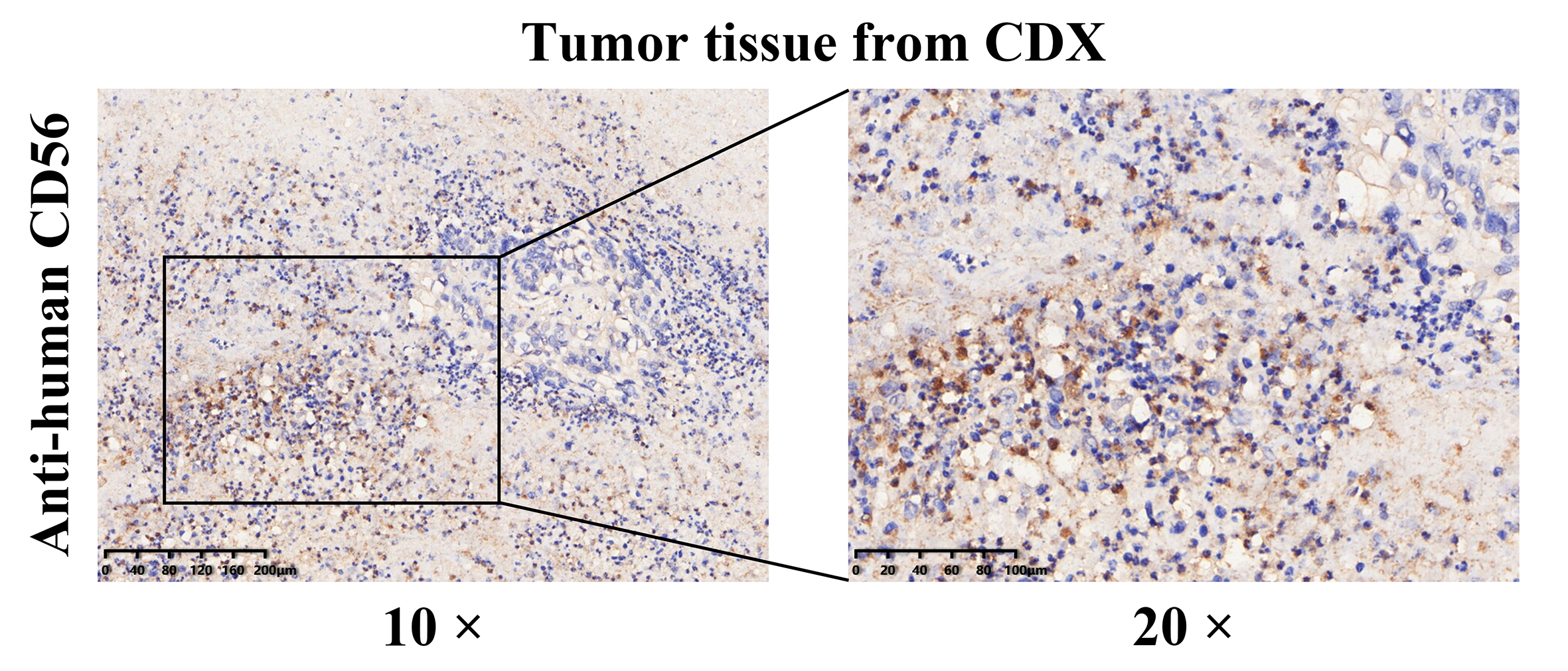

Supplement: Supplementary Figure 5 — The iPSC-derived MUC1-targeted CAR-NK cells significantly prolonged the survival of OTSCC xenograft BNDG mice. The survival curves were obtained from three groups of BNDG mouse OTSCC (MUC1 positive) xenograft models. These groups were respectively injected with CAR-iPS-NK cells, iPS-NK cells, and the blank control (saline). Through weekly monitoring for a duration of three months, we observed that MUC1-CAR-NK cells significantly prolonged the survival of mice. Intriguingly, neither the iPS-NK cells nor CAR-iPS-NK cells induced any mortality in the mice, as evidenced by their noticeably longer survival compared to the blank control (saline) group. [file Image_5.jpg]

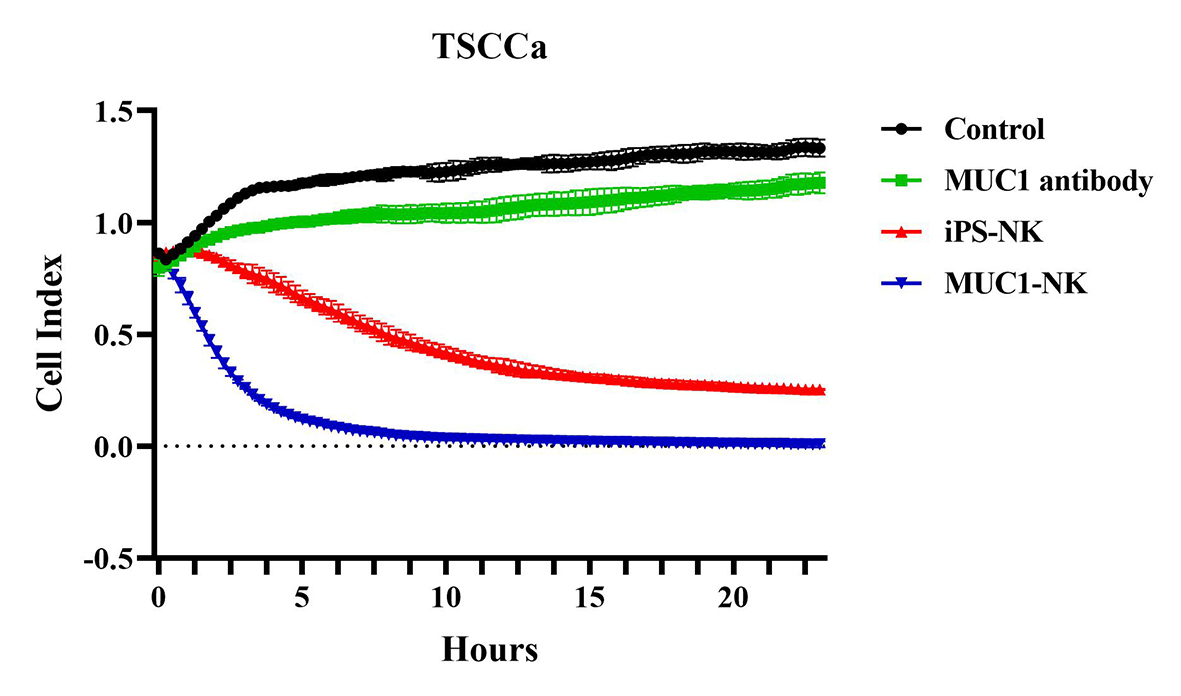

Supplement: Supplementary file 6 [file Image_6.jpg]

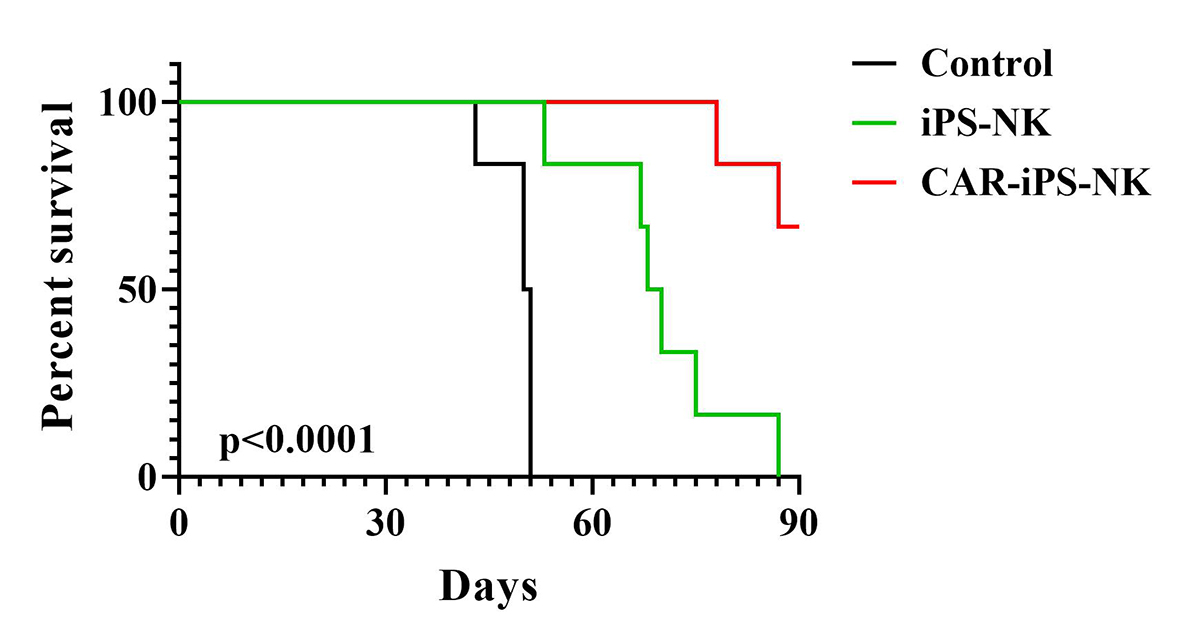

Supplement: Supplementary file 7 [file Image_7.jpg]
